# Mechanisms protecting host cells against bacterial pore-forming toxins

**DOI:** 10.1007/s00018-018-2992-8

**Published:** 2018-12-27

**Authors:** Cláudia Brito, Didier Cabanes, Francisco Sarmento Mesquita, Sandra Sousa

**Affiliations:** 1 i3S-Instituto de Investigação e Inovação em Saúde, IBMC, Universidade do Porto, Rua Alfredo Allen, 208, 4200-135 Porto, Portugal; 2 Programa Doutoral em Biologia Molecular e Celular (MCbiology), Instituto de Ciências Biomédicas Abel Salazar, Universidade do Porto, Rua Jorge de Viterbo Ferreira 228, 4050-313 Porto, Portugal; 3 Present Address: Global Health Institute, School of Life Science, Ecole Polytechnique Fédérale de Lausanne, Lausanne, Switzerland

**Keywords:** Pore-forming toxins, Cholesterol-dependent cytolysins, Plasma membrane damage, Plasma membrane repair, Blebbing, Shedding, Actomyosin remodeling, Host signaling

## Abstract

Pore-forming toxins (PFTs) are key virulence determinants produced and secreted by a variety of human bacterial pathogens. They disrupt the plasma membrane (PM) by generating stable protein pores, which allow uncontrolled exchanges between the extracellular and intracellular milieus, dramatically disturbing cellular homeostasis. In recent years, many advances were made regarding the characterization of conserved repair mechanisms that allow eukaryotic cells to recover from mechanical disruption of the PM membrane. However, the specificities of the cell recovery pathways that protect host cells against PFT-induced damage remain remarkably elusive. During bacterial infections, the coordinated action of such cell recovery processes defines the outcome of infected cells and is, thus, critical for our understanding of bacterial pathogenesis. Here, we review the cellular pathways reported to be involved in the response to bacterial PFTs and discuss their impact in single-cell recovery and infection.

## Introduction

The plasma membrane (PM) constitutes a selective barrier between the intracellular and extracellular environment, defining the limits of every living eukaryotic cell [1, 2]. Its emergence was a critical event during evolution, and its integrity is crucial to maintain cellular homeostasis and support life [3]. Transient PM lesions still occur in healthy conditions, particularly, in tissues under high mechanical stress or biochemical stress such as muscle, skin, or gut epithelia [4, 5]. Conversely, persistent PM damage hallmarks several pathologies, such as heart failure, neurodegeneration, and infection [6–9].

Being the first protective cellular barrier, the PM is preferentially targeted by pathogens to exploit host intracellular nutrients, disrupt signaling, cross-tissue barriers, and/or kill immune cells [10–14]. In particular, bacteria secrete monomeric pore-forming toxins (PFTs), which oligomerize upon binding to the host PM and assemble into transmembrane stable pores that permeabilize cells to ions, metabolites, and proteins [11, 15–21], triggering a variety of coordinated host-cell responses. PFTs are both necessary and sufficient for the pathogenesis of several bacterial species [22–24]. Such proteins exist in virtually all the kingdoms of life, comprising different structural families, for which the mechanisms of oligomerization and pore formation have been extensively characterized (reviewed in [15]). In contrast, their effects at the cellular level and specific roles in disease development are by far less understood.

PM injury elicits multiple responses depending on the nature of the damage and the cell type involved. Despite certain specificities, these responses are based on conserved events that include sensing the damage, activating repair mechanisms, restoring homeostasis, and activating innate immunity, thereby alerting neighboring tissues. In recent years, considerable advances were made regarding the identification and characterization of PM repair mechanisms and single-cell recovery processes. Such studies have highlighted the sequential nature that underlies the spatio-temporal coordination of cell recovery, but have predominantly focused on localized mechanical- or laser-induced damage (reviewed recently in [5, 25]). Accordingly, although the mechanisms identified are still relevant in the context of PFT-mediated damage, the specific features of repairing stable protein pores and recovering cellular homeostasis following PM damage caused by bacterial PFTs remain poorly understood. Nevertheless, here, we review the large amount of work carried during the past years, concerning the multitude of cellular responses to various bacterial PFTs. We attempt to discuss such responses in light of our current understanding of general PM repair mechanisms and single-cell recovery mechanisms to provide a more complete view of the processes deployed by host cells to specifically face PFT-mediated intoxication. When adequate, we also discuss their relevance during infection.

## Sensing the damage

**Fig. 1:**
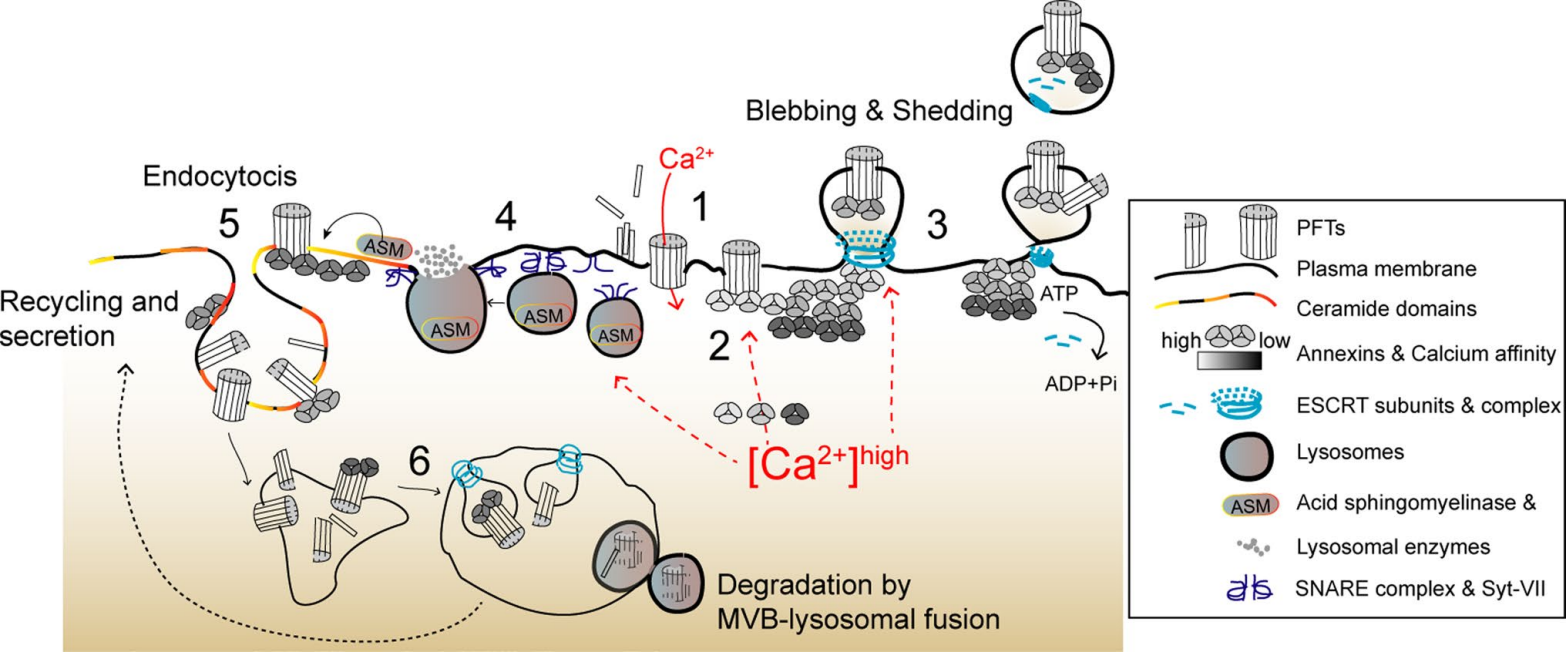
Proposed overview model depicting calcium-dependent PM repair mechanisms that protect host cells against PFTs. (1) Toxin oligomerization, pore formation, and calcium influx initiate the activation of calcium-dependent protective events. (2) Annexins are recruited to damaged areas according to their differential calcium sensitivity (gray scale) and assemble into 2D protein arrays to clog PM pores. (3) PM blebbing and shedding occur at damaged sites and involve recruitment of ESCRT subunits, which facilitate budding and ATP-dependent release of PM vesicles containing PFT pores, annexins, and ESCRT components. (4) In parallel, calcium influx triggers PM docking and exocytosis of cortical lysosomes. Upon assembly of SNARE complexes, the calcium sensor Synaptotagmin VII (Syt-VII) enables fusion of lysosomes with the PM and release of lysosomal enzymes, in particular ASM. (5) ASM hydrolyses PM sphingomyelin, producing ceramide domains, which facilitate membrane invagination and endocytosis of PFTs’ pores and incomplete pore structures. Ceramide domains may also contribute to annexin recruitment. (6) PFTs traffic to MVBs through ESCRT-dependent sorting and are degraded via MVB–lysosomal fusion. Toxins may also be recycled back to the PM and further secreted

**Fig. 2:**
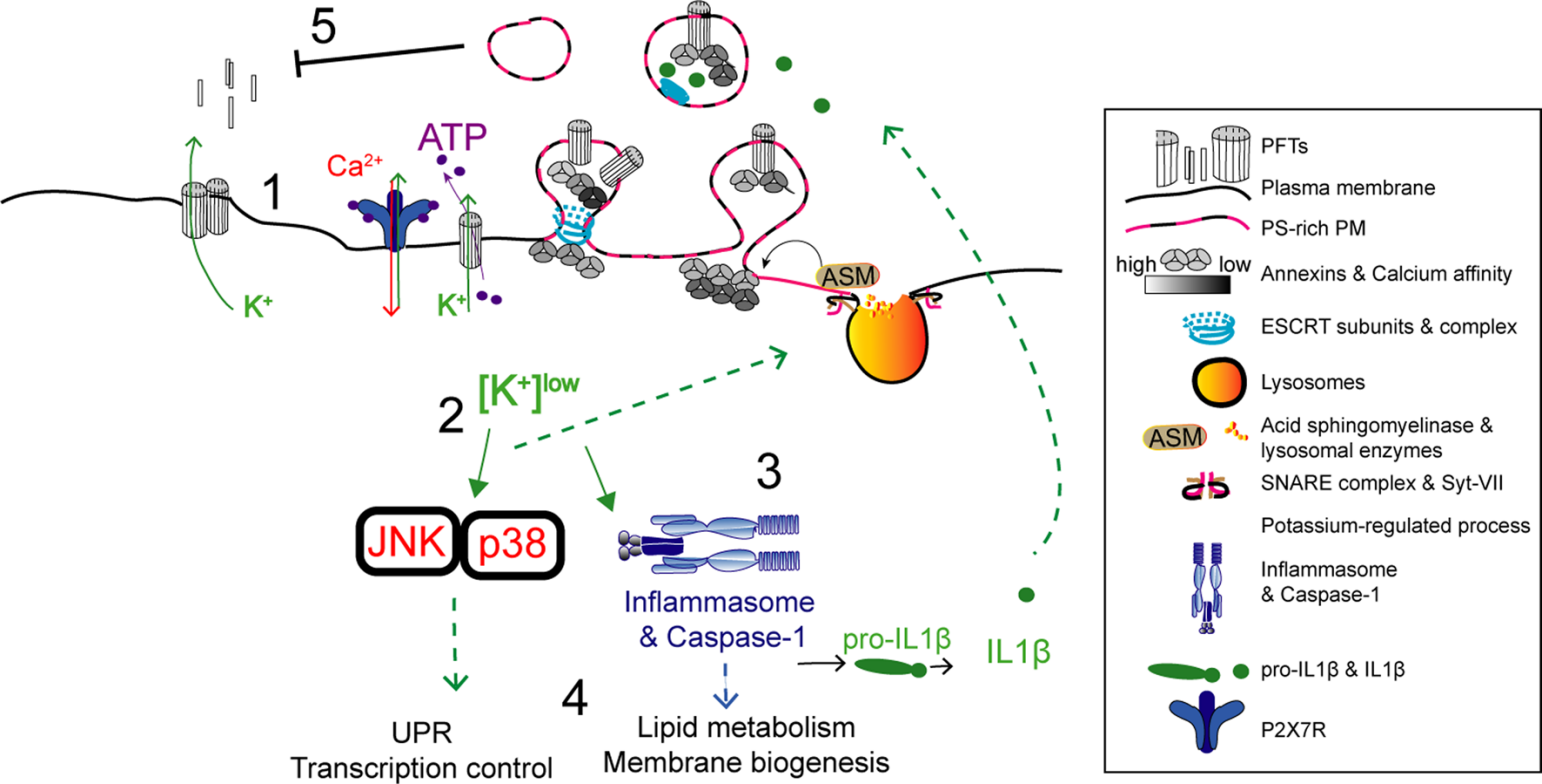
Proposed model for potassium-dependent host-protective mechanisms against PFT intoxication. (1) Potassium efflux and release of ATP occur across PFT-assembled pores. Extracellular ATP activates the P_2_X_7_ receptor and cation channel, triggering further potassium efflux and calcium influx. These events activate several recovery processes that include: (2) PM translocation of lysosomal ASM, subsequent remodeling of PM lipid composition, and release of phosphatidylserine (PS)-enriched PM vesicles. This process may enable the secretion of cytokines and occurs downstream activation of p38. (3) Inflammasome activation, caspase-1 processing and activation of IL-1 beta secretion. Caspase-1 activation increases lipid metabolism and membrane biogenesis pathways. (4) Potassium efflux also activates MAPK signaling, in particular p38 and JNK, which further control the UPR and protective transcriptional responses required for survival against PFTs

**Fig. 3:**
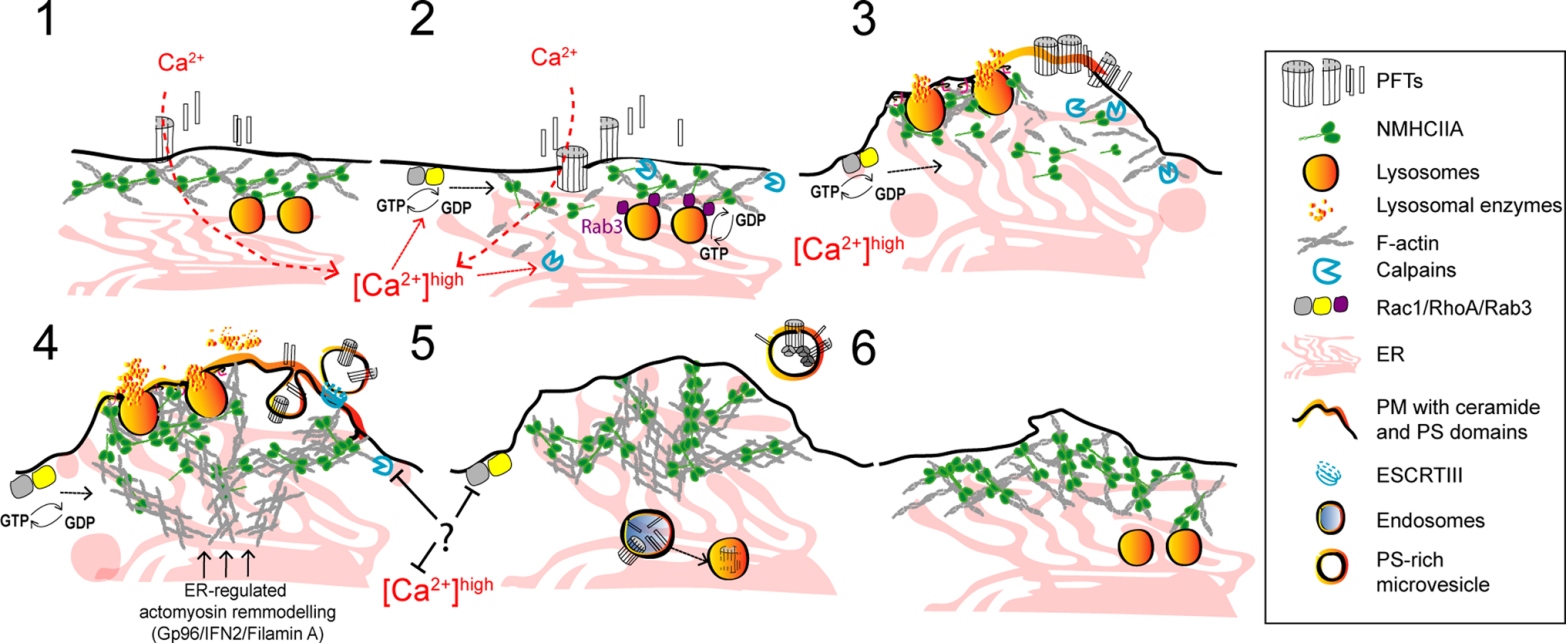
Proposed model illustrating the protective mechanisms of actomyosin remodeling in response to PFT intoxication. (1, 2) Pore formation and the subsequent calcium influx induce cortical actomyosin remodeling by: disassembling actomyosin structures; activating the GTPases Rac1 and RhoA, and enhancing calpain activity which breaks cytoskeletal–PM contacts and disrupts interactions between actin and actin-binding proteins. Cortical lysosomal positioning is maintained by interactions between Rab3A and NMIIA. The rise in cytosolic calcium activates Rab3 and promotes actin remodeling contributing to binding, docking, and fusion of cortical lysosomes with the PM. (3) Actomyosin remodeling and lysosome secretion lower PM tension and modify the PM lipid composition causing PM blebbing, ruffling, and shedding or internalization of PFT pores. Actomyosin reorganization is regulated by RhoA and Rac1 and is stabilized by the formation of NMIIA cortical bundles. Concomitant ER expansion and ER–cytoskeletal interactions also contribute to stabilize the cortical actomyosin network. (4) Following shedding or internalization of PFT pores, cells re-establish cytoskeletal organization and recover normal cytosolic calcium levels

PM disruption allows the influx of calcium and efflux of potassium, altering the intracellular-ion composition, which has long been recognized as the primary trigger for cell responses to PM damage (Figs. 1, 2) [5, 25, 26]. The influx of extracellular calcium activates PM repair pathways (Fig. 1) and protective cytoskeletal remodeling (Fig. 3) [27–30]. However, overwhelming and long-lasting calcium elevations (approximately > 20 µM) are toxic [31, 32] and compromise host-cell signaling, which can desensitize immune cells [33], destabilize tissue barriers [34], or induce cell death [35–37]. Thus, controlling the rise of cytosolic calcium levels appears fundamental to determine cell fate following PM disruption, either promoting survival or triggering cell death [32, 38, 39].

Potassium efflux promotes stress-activated and mitogen-activated protein kinase (MAPK) pathways that can protect against PFT activity in vitro and in vivo [40–43], but the drop in cytosolic potassium levels also alters the cellular metabolic state, triggers innate immune signaling, and may cause pro-inflammatory cell death (Fig. 2) [35, 41, 44].

The magnitude of ion imbalance depends on the dimension of the PM wound which, in the case of toxin-induced pores, varies greatly according to the stoichiometry and size of the pore [15]. Cholesterol-dependent cytolysins (CDCs), such as listeriolysin O (LLO) and streptolysin O (SLO), secreted by the Gram-positive bacteria *Listeria monocytogenes* and *Streptococcus pyogenes,* respectively, assemble into large (30–50 nm in diameter) heterogeneous pores, whereas smaller toxins, such as aerolysin from *Aeromonas hydrophila* or alpha-toxin from *Staphylococcus aureus*, originate pores of only ~ 2 nm in diameter [15].

The full recovery of PM integrity was shown in different cell types perforated by small or large PFTs [31, 41, 45], yet counter-intuitively, cells damaged by small toxin pores, appear to take longer to recover PM integrity (~ h), when compared to large CDC-induced damage (~ min). This has been largely attributed to the lower calcium permeability of small pores, and the consequent defect to efficiently trigger rapid calcium-dependent repair mechanisms. The recovery from damage caused by small toxin pores must rely mainly in potassium-dependent cellular responses, which, indeed, protect cells upon attack by a variety of small PFTs such as alpha-toxin [46], *Vibrio cholerae* cytolysin (VCC) [47, 48], and Cry5B [48]. In agreement with these observations, mutations that increase the channel width of the small PFT phobalysin from *Photobacterium damselae*, enhanced calcium-dependent repair, whereas bulky amino acid residues within VCC channels were shown to delay the recovery [49].

Not only the size of the pore, but also its structure, may determine the specificity of repair pathways. Damage caused by Sticholysin II, a small actinoporin produced by a sea anemone, is repaired with equivalent kinetics of CDCs [50]. CDCs originate both stable protein-lined and heterogeneous pores, which, at low stoichiometry, form less stable arc-like structures composed by proteins and lipids siding each other [51–54]. Comparably to such less stable CDC structures, actinoporins form intercalated protein–lipid pores [55, 56], also less stable than protein-lined pores formed by other small toxins [15, 51, 52]. Thus, heterogeneous pore-forming structures may be repaired more efficiently, as it occurs with electroporation-induced lipid wounds, indicating that the nature of the pore also determines its effective repair.

Alteration of the cytosolic ion composition upon PM damage relies on additional secondary events: certain PFTs can induce the opening of intracellular calcium stores [33] and allow the release of ATP to the extracellular environment. Host cells recognize extracellular ATP, which further enhances intracellular-ion imbalance [57], but may also trigger protective responses in neighboring cells (Fig. 2) [58–60]. These processes rely on the expression of P_2_X_7_ receptor (P_2_X_7_R), which is a cation channel. However, given the broad range of ATP sensitivities displayed by purinergic receptors [61, 62], it is possible that alternative P_2_X receptors also respond to ATP released from PFT-damaged cells.

Finally, the endogenous pore-forming activity of host proteins such as mixed lineage kinase-like (MLKL) or gasdermin D may also contribute to further promote ATP and potassium efflux or the influx of extracellular calcium [63]. Gasdermin D and MLKL form pores in the inner PM leaflet, during pro-inflammatory cell death mechanisms (pyroptosis and necroptosis, respectively), which can be activated by PFTs’ intoxication [35, 64]. Specific roles for gasdermin D or MLKL during cellular recovery from PFT intoxication have not been established. Nevertheless, activation of crucial pyroptotic and necroptotic effectors during PFT intoxication enhances alarmin release and promotes host inflammation during pneumonia caused by *Serratia marcescens* infection [64].

Overall, host responses to PFT intoxication will vary depending on the differential cytosolic ion gradients produced by structurally different PFTs, their concentration, and the release of additional cellular metabolites.

## Plasma membrane repair

### Clogging the pore

The influx of extracellular calcium following PM damage promotes the exocytosis of cortical vesicles (e.g., lysosomes) and the recruitment of protein arrays to PM wounds. These processes, through the formation of a patch of homotypically fused vesicles and a clog of fusogenic protein arrays, were proposed to limit the loss of cytosolic content and the rise of intracellular calcium to toxic levels during mechanical- or laser-induced PM damage [5]. Such calcium-mediated exocytosis also reduces membrane tension, which may contribute to the spontaneous resealing of lipid-based wounds [65]. However, stable protein pores, such those generated by PFTs, do not spontaneously reseal and must be actively removed.

Annexins, one of the major components of clogging protein complexes, are cytosolic calcium sensors with the capacity to aggregate, bind phospholipids, and promote membrane fusion in a calcium-regulated manner [66, 67]. They are promptly recruited to PM lesions in cells damaged by different CDCs (SLO and pneumolysin, PLY) [31]. Upon pore formation, annexins sequentially and reversibly translocate to the PM surface according to their different calcium sensitivities (Fig. 1) [68]. Annexins with high calcium sensitivity (A2 and A6) are early recruited to the sites of PM damage, and were detected in PM blebs and vesicular or tubular structures released by SLO- or PLY-intoxicated cells [68, 69]. In turn, annexins with low calcium sensitivity (A1 and A5) appear later around PM wounds and their translocation to the PM surface correlates with the inability of cells to recover from PM damage [68], presumably because the intracellular calcium concentration has reached a toxic threshold (~ 20 µM). Annexins (A2, A6, A1, and A5) exhibit protective roles upon mechanical- or laser-induced PM damage and in PM damage-related disorders [67]. Yet, how annexins clog a protein pore and protect cells during PFT intoxication remains unclear. Nevertheless, A1 localizes to PFT-damaged PM regions and is detected within large PM blebs that appear to compartmentalize cytoplasmic content. Moreover, similarly to what was observed upon mechanically induced damage of HeLa cells [70], A1 depletion or targeting with blocking antibodies increases susceptibility to CDCs, thus confirming a protective role against PFTs [71, 72]. Furthermore, cryo-electron tomography of vesicles released by PLY-damaged cells show high-density structures concentrated below toxin pores, resembling the A5 two-dimensional arrays that assemble at sites of laser-induced PM wounds [69, 73–75]. Mass spectrometry analysis confirmed that such vesicles are enriched in annexins [69]. Altogether these observations led to speculate that annexins assemble into two-dimensional arrays that clog PFT pores, avoiding the detrimental diffusion of calcium to the entire cell (Fig. 1) [32, 68, 69]. Such clog may also isolate damage within PM blebs [72].

### Quarantining PM damage: blebbing

Blebbing is a universal cellular response to PM injury described in different processes such as cytokinesis, cell migration, and apoptosis [76, 77]. PM blebs require calcium-dependent actomyosin contraction and result from the disruption of PM–cytoskeleton interactions, which decreases PM tension and enables its expansion. Since vesicle exocytosis also reduces PM tension, it is possible that this process contributes to blebbing upon PM damage. Intriguingly, in the context of PFTs, blebbing may result from intrinsic properties of specific PM lipid domains that respond to toxin oligomerization and binding, before PM disruption [78, 79].

**Fig. 4:**
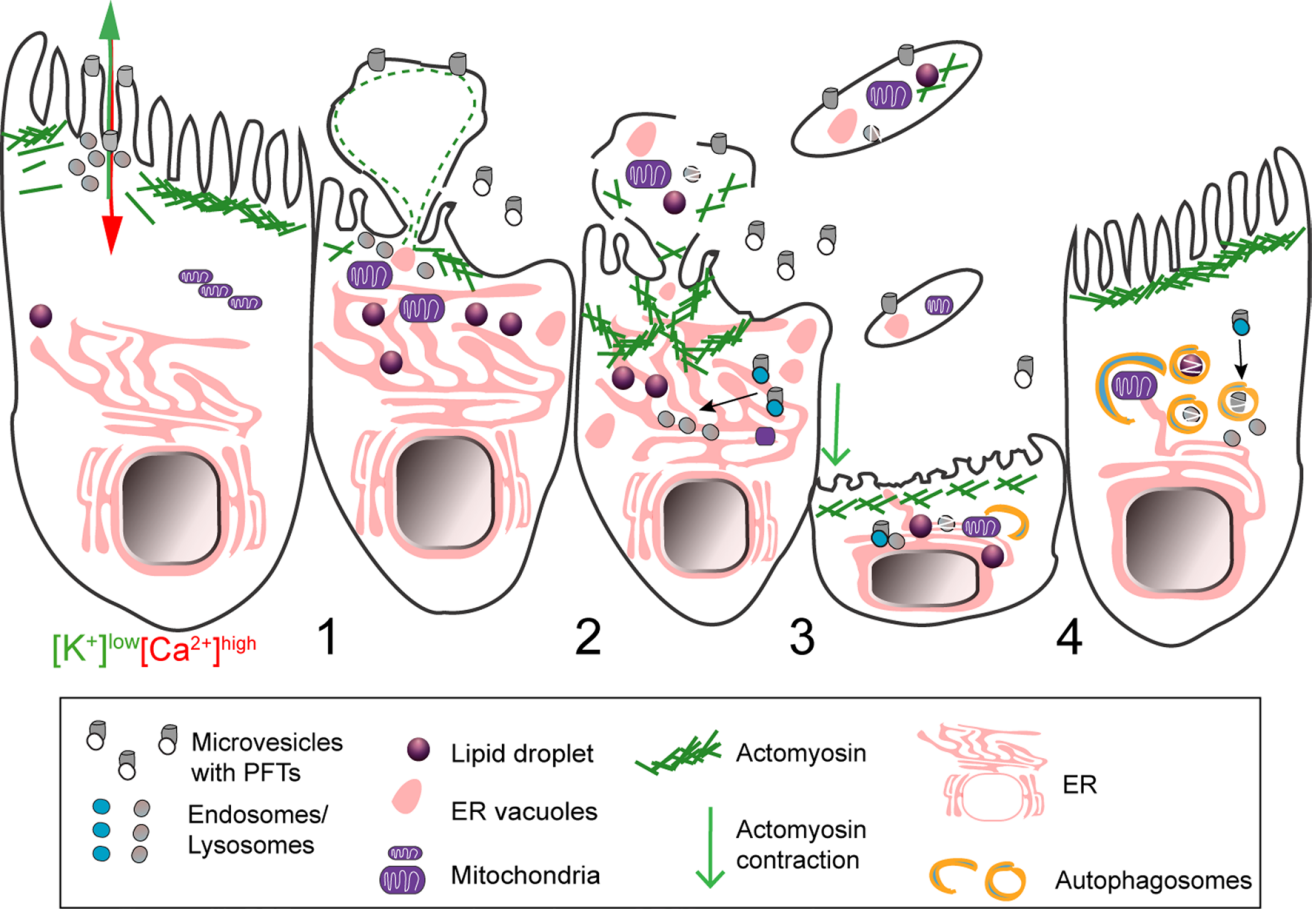
Schematic representation of shedding of large cytoplasm-containing blebs or extrusions, and thinning of epithelia damaged by PFTs. (1) The ion imbalance generated by pore formation promotes apical actomyosin remodeling and vesicle secretion. (2) Both processes lower PM tension contributing to PM blebbing, remodeling, and shedding. The cytosolic ion imbalance alters organelle dynamics and causes organelle damage, including: lipid-droplet formation, mitochondria fission and enlargement, ER expansion and vacuolation, lysosomal secretion and rupture. (3) Damaged organelles are detected in the proximity of the PM and within cytoplasmic extrusions or large cell particles (e.g., blebs and villi) released by intoxicated cells. (4) Epithelial integrity and cellular homeostasis are maintained by transient (~ h) contraction and thinning of the epithelial actomyosin network and removal of damaged organelles via autophagy. Toxin pores are eliminated by PM shedding, endocytosis, and autophagic targeting

**Fig. 5:**
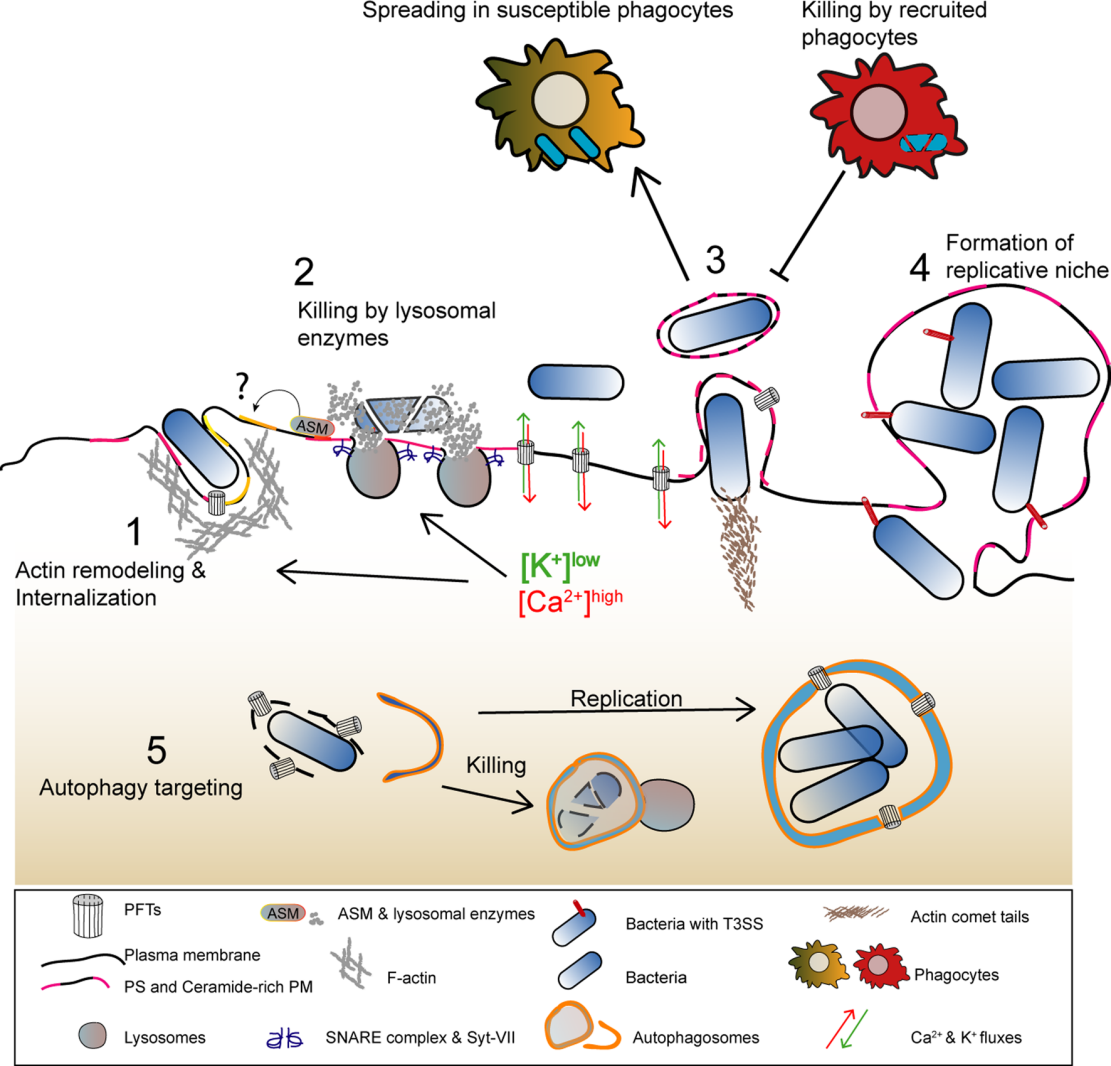
Model illustrating how the main host-protective responses to PFT intoxication influence the outcome of bacterial infections. (1) Lysosome exocytosis and actomyosin remodeling promote the secretion of lysosomal enzymes that alter PM lipid composition and enable activation of endocytic pathways which allow pathogen internalization. (2) The release of hydrolytic enzymes contributes for pathogen killing. (3) Alterations in PM tension caused by PFT-induced damage promote the formation and release of PM protrusions and/or blebs that allow the dissemination of *L. monocytogenes* in enclosed vesicles or allow the shedding of intracellular bacteria. Released bacteria-containing vesicles can also be subsequently engulfed and killed by recruited phagocytes (efferocytosis). (4) Large PM blebs may sustain the replication of intracellular *P. aeruginosa.* (5) Autophagy targets PFT-producing bacteria in the host cytosol, upon vacuolar escape, and either promotes pathogen killing or the formation of SLAPs, a niche for *L. monocytogenes* replication

In PLY- or SLO-damaged cells, large PM blebs were proposed to create a confined space where calcium concentration is higher than in the cell body [31, 72]. Large blebs possibly protect the cell from deleterious calcium elevations and loss of cytosolic content. The majority of large PM blebs retract, supporting their role as clogging structures or, alternatively, as secondary events of the PFT-induced cortical cytoskeletal disruption. Nonetheless, different cell types were shown to release large (µm size) blebs, villi, or bleb-like structures containing cytoplasmic material in response to PM damage caused by protein pores (Fig. 4) [69, 80, 81]. Such release of large blebs may result from the inability of cells to repair overwhelming damaged areas and/or derive from the engagement of cell death pathways. Along with the shedding of smaller blebs, the release of large PM structures will likely allow the removal of PFT pores, the disposal of irreversibly damaged organelles, and also convey cytoplasmic inflammatory signals. Indeed, blebs produced by apoptotic cells carry and release cytoplasmic danger signals [82], and large cell particles shed by cancer cells, in vivo, are delivered to myeloid cells, thereby altering their behavior [83]. *Drosophila melanogaster* gut epithelial cells targeted by *S. marcescens* expressing the PFT ShlA also release bleb-like cytoplasmic extrusions containing damaged organelles [84]. This response maintains epithelial integrity and limits host injury, as flies infected with ShlA-deficient strains undergo amplified injury. Similarly, cytoplasm-containing blebs from *Mycobacterium*-infected cells also deliver intracellular bacteria to phagocytic cells (Fig. 5) [85]. On the other hand, bacteria can hijack PM blebs to promote dissemination. This is the case of *Pseudomonas aeruginosa* that utilizes type III secretion system (T3SS) pore-forming translocon proteins to induce PM blebbing and exploit blebs as niches for replication [86]; and of *L. monocytogenes,* which promotes LLO-dependent blebbing to support cell-to-cell spread within PM blebs (Fig. 5) [87].

### Removing PM pores: shedding

PM blebbing enables the shedding of microvesicles, which is regarded as a major PM repair mechanism that protects from detergent-induced or mechanically induced wounds and against intoxication with small stable PFTs, such as alpha-toxin or Cry5B, and large CDCs [32, 69, 79, 88–92]. Elimination of PFTs within PM vesicles occurs in various cell types and may constitute an intrinsic protection mechanism, which is further enhanced by toxin oligomerization, pore formation, and calcium entry [79, 92]. This mechanism was recently proposed to benefit from the heterogeneous binding of certain PFTs to cholesterol-rich PM domains [93], and allows the rapid shedding of fully formed pores and incomplete pore-forming structures within small PM microvesicles (~ 100 to 200 nm) [69, 92]. PM shedding occurs at wound sites, and involves passive PM blebbing, active vesicle budding, and release of vesicular structures enriched in endosomal sorting complex required for transport (ESCRT) proteins, annexins, PFT pores, and other molecules (Fig. 1) [69, 78, 79, 90, 92]. ESCRT complexes have established roles in membrane deformation and scission processes [e.g., multivesicular body (MVB) biogenesis and viral budding], and ESCRT-III-mediated PM shedding was fully characterized in laser-wounded cells [88]. In this context, ESCRT-III adaptor proteins, the apoptosis linked gene (ALG)-2, and ALG-2-interacting protein X (ALIX) enable the rapid (~ min) calcium-dependent recruitment of ESCRT subunits to PM wounds [94]. The adenosine triphosphatase Vps4, responsible for ESCRT-III disassembly, is recruited subsequently and contributes to the budding and ATP-dependent pinching of PM microvesicles containing lesions, thereby protecting cells from small (< 100 nm) PM wounds [88, 94, 95]. In agreement, LLO-intoxicated cells show punctate distribution of the ESCRT-III component CHMP4B at the plasma membrane [88], and vesicles released by cells challenged with different CDCs are enriched in ESCRT assembly and disassembly subunits [69].

Interestingly, ALG-2 can interact with different annexins in vitro [96], and the recruitment of annexin A1 to the PM of mechanically or laser-damaged cancer cells is followed by PM shedding [97], suggesting an interplay between clogging and shedding PM injuries. The ESCRT-III complex also coordinates blebbing and shedding of PM vesicles promoted by MLKL activity during necroptosis [98], the major pro-inflammatory programmed cell death pathway promoted by PFTs in vivo [35]. Hence, ESCRT proteins have emerged as important mediators of cell-autonomous defenses and innate immunity against bacterial pathogens. However, in contrast to the well-established role of ESCRT proteins in viral budding, few reports assessed ESCRT-mediated repair during bacterial infections. Nonetheless, PM shedding occurs in vivo upon PFT intoxication, concomitantly with the other PM repair processes [48].

Extracellular ATP, which can be released by PFT-damaged cells, also triggers PM blebbing and shedding, via the activation of P_2_X_7_R channels, potentially protecting neighboring cells prior PFTs’ attack [99–101]. In line with these observations, artificial liposomes can reduce toxin binding to host cells in vitro and protect against PFT-dependent infections in vivo [102, 103]. Thus, PM shedding not only represents a major cell-autonomous defense but may also prevent recurrent toxin attacks by trapping free toxin molecules within secreted vesicles [72].

### Removing PM pores: lysosome exocytosis and endocytic degradation

As mentioned above, PM ruptures trigger calcium-dependent exocytosis of peripheral vesicles, predominantly lysosomes [90, 104]. These vesicles were proposed to patch PM wounds [65] and reduce PM tension, contributing to the spontaneous resealing of lipid-based injuries, such as laser-induced or mechanically induced PM wounds [105]. Lysosome exocytosis upon PM damage, is reminiscent to the secretion of granules by cytotoxic lymphocytes or professional secretory cells and involves calcium-dependent interactions between the calcium sensor synaptotagmin VII, dysferlin, and lysosomal (e.g., VAMP-7) and PM (e.g., SNAP-23 and syntaxin-4) SNARES (N-ethylmaleimide-sensitive factor attachment protein receptors) (Fig. 1) [106–109]. In addition, small GTPases (Rab3, Rab10, and Arl8b), the Rab3 effector synaptotagmin-like protein 4a (Slp4-a) and the actin motor protein non-muscle myosin IIA (NMIIA), also control cortical lysosome positioning required for PM repair (Fig. 3) [110, 111]. Secretion of lysosomes allows the release of lysosomal enzymes, such as acid sphingomyelinase (ASM), and cathepsin B and L, which alter local PM composition promoting PFT removal via endocytosis (Fig. 1) [112–114]. In particular, ASM was shown to hydrolyze sphingomyelin into phosphorylcholine and ceramide domains, triggering the endocytosis of PFT pores within small (50–100 nm) lipid-rich PM invaginations termed caveolae [115–117]. Caveolin- or ASM-deficient cells have impaired ability to repair PFT-induced injuries [104, 118, 119] and extracellular ASM is sufficient to promote SLO internalization [104, 115]. However, to date, internalization of active pores by caveolar-dependent mechanisms has never been directly visualized and the role of endocytosis as a mechanism of PFT removal remains controversial. Indeed, blocking endocytosis does not compromise the removal of PFT pores from intoxicated cells [79]. In addition, endocytosed PFT pores would remain associated with the membrane of endosomes possibly leading to endosomal leakage and release of toxic enzymes to the cell cytosol. Nevertheless, it has been shown that toxin internalization occurs for various PFTs [112, 120, 121], which apparently are sorted to MVBs in a ubiquitination/ESCRT-dependent manner. Accordingly, poly-ubiquitinated proteins are observed close to laser-induced PM wounds in parallel to PM shedding [88]. PFTs are possibly degraded upon MVB–lysosomal fusion [112, 120] or can be re-routed to the extracellular milieu within exosome-like compartments (Fig. 1) [120]. Moreover, it has been increasingly clear that the endocytic machinery contributes to the survival of cells upon intoxication. Endocytic components such as caveolin-1 and GRAF1 were recently shown to play a role during PM repair of PFT pores by acting on the remodel and regeneration of the normal composition of the PM following repair [122]. Based on ultra-structural evidences, a recent report proposed a model where PFT-induced active pores are removed by the shedding of microvesicles, whereas endocytosis restores PM homeostasis by removing inactive PFT monomers and vesicles that failed to shed, once repair is complete [92].

Lysosome exocytosis may be linked to PM shedding. In glial cells, secretion of ASM promotes PM shedding of phosphatidylserine (PS)-rich vesicles downstream P_2_X_7_R activation (Fig. 2) [123]. This process requires Src kinase-dependent phosphorylation of the p38 MAPK [123] that is activated by all the tested PFTs and promotes the shedding of cell adhesion molecules in response to *S. aureus* PFTs [124]. Thus, it is possible that p38 activation per se explains the removal of small PFT pores, which do not trigger massive calcium-dependent repair. Yet, further work is required to address this hypothesis.

Off note, ASM-produced PM ceramide domains increase annexin A1 binding to the PM [125]. Thus, annexin clogging, PM shedding, lysosomal exocytosis, and further endocytosis of PFTs may likely benefit from the coordinated action of common molecular effectors which, altogether, protect upon intoxication by various PFTs. In agreement, increased endocytosis and PM shedding occur during *Caenorhabditis elegans* intoxication with different PFTs, through a process that depends on the small GTPases Rab5 and Rab11 [48], master endo- and exocytic regulators, respectively.

Fusion of cortical lysosomes with the PM and the consequent extracellular delivery of lysosomal enzymes is not only important for PM repair, but was also proposed as a conserved cell-autonomous defense against pathogens that damage the host-cell PM (Fig. 5) [106, 126]. In favor of such mechanism, Arl8 and NMIIA, which promote calcium-dependent lysosome exocytosis, contribute to bacterial clearance in vivo, or can reduce host-cell invasion by bacterial pathogens, respectively [110, 127]. Concurrently, *Mycobacterium tuberculosis* diminishes the expression of synaptotagmin VII to promote PM damage and necrotic-based dissemination [128].

Endocytosis following PM perforation can also be exploited by different pathogens to induce internalization [129, 130]. In fact, LLO is sufficient to promote calcium- and potassium-mediated bacterial internalization and endocytosis of inert beads [129, 131]. In the other hand, avoiding PFT-mediated cytotoxicity can also favor the pathogenesis of intracellular bacteria. In this context, it was recently shown that a specific C-terminal PEST-like sequence present in LLO mediates its removal from the inner face of the PM by endocytosis, preventing premature killing of infected cells, thereby favoring *L. monocytogenes* infection [132].

Altogether it is becoming evident that clogging of the pore, PM blebbing, shedding of PM vesicles, and lysosomal exocytosis and endocytosis are interconnected mechanisms that cooperate during PM repair upon PFTs attack, and determine the fate of intoxicated cells.

## Intracellular responses

### Cytoskeleton remodeling

Regardless the source of PM damage, cells undergo dramatic cytoskeletal alterations, which result from mechanical or biochemical cues that strongly affect the homeostatic tension properties of the cell [39, 133]. The remodeling of the actomyosin cytoskeleton reduces PM tension and facilitates vesicle recruitment and PM shedding, thus promoting PM repair [90, 134–137]. Accordingly, compounds that stabilize the actomyosin network, such as jasplakinolide and phalloidin, hinder the recovery of PM integrity upon mechanical-, laser-, and PFT-induced damage [90, 134, 138, 139], whereas actin-depolymerizing agents (e.g., cytochalasin D, latrunculin, and DNAse I) lead to faster repair [90, 105, 134, 135, 140, 141].

Although a direct interaction between PLY and actin has been reported [142], the targeting of the actomyosin cytoskeleton by PFTs occurs mainly through the sustained rise in cytosolic calcium levels caused by PFT-mediated damage. This process induces the activation of different cytoskeleton-modulating enzymes, which is sufficient to trigger actin cytoskeleton remodeling, facilitating cell adaptation, and recovery responses to different types of PM damage (Fig. 3) [143]. In particular, the calcium-dependent cysteine proteases [144], calpains, favor repair of mechanical and laser injuries [145–147], facilitate vesicle fusion with the PM [146], and can cleave actin-associated proteins ultimately leading to actin remodeling [144, 148]. In LLO-damaged cells, calpain-2 is recruited to cortical actomyosin bundles, which assemble around sites of PM damage and blebbing [89]. Moreover, during *Streptococcus pneumoniae* infection, PLY-dependent calpain activation promotes the release of the pro-inflammatory cytokine IL-1 beta by the infected macrophages [149], a process that can occur via PM shedding [59]. Interestingly, an evolutionary and biochemical link between ESCRT proteins and calpains has been proposed, which may have crucial roles during yeast adaption responses [150, 151].

The assembly and dynamics of actomyosin rings that provide purse-string forces to close laser-induced PM and epithelial wounds, in *Xenopus* oocytes or *Drosophila* embryos, involve the coordinated activity of Rho GTPases and NMII [138, 141, 152]. Rho GTPases are also activated during PLY intoxication of neuronal cells and promote calcium-dependent actin remodeling (Fig. 3) [153]. Pharmacological inhibition of actin polymerization delays the formation of an A6 PM clog in laser-wounded muscle cells [154], indicating a role for actin remodeling during PM clogging. Whether this occurs upon intoxication with PFTs is still unclear.

Nevertheless, NMIIA rearrangements and the assembly of stable cortical actomyosin bundles at sites of PM blebbing and PFT-induced damage correlate with increased cell survival following LLO intoxication [89]. This process involves the translocation of endoplasmic reticulum (ER) proteins to the cell surface and depends on the ER chaperone Gp96 [89]. Gp96 interacts with NMIIA and the actin adaptor Filamin A [89], and regulates NMIIA activity and cytoskeletal remodeling in response to PM damage [89]. Remarkably, both Gp96 and NMIIA protect cells against PFTs and Gp96 is critical for host survival during LLO-dependent *L. monocytogenes* infection [89].

Filamin A and Gp96 appear to coordinate actin organization through processes that may rely on ER–cytoskeleton interactions, ER dynamics, and polarized secretion [89, 155–157]. Of note, polarized lysosome secretion is controlled by NMIIA and is essential for PM repair of mechanically, laser-, or PFT-induced wounds [111, 136, 158]. The relevance of generalized actomyosin reorganization during PFT intoxication is unclear. However, a similar response was proposed to underlie acute morphological adaptations to PM damage, cell stress, or migration [143]. This mechanism is regulated by calcium and the ER-associated formin, IFN2, and involves IFN2-mediated turnover of cortical actin filaments and concurrent polymerization from the ER [143]. Altogether, these observations suggest a strong link between cytoskeleton remodeling, ER dynamics, and vesicle trafficking, supporting the complexity of PM repair pathways.

Actomyosin remodeling may also be important for epithelial integrity. During cytoplasmic extrusion, triggered by bacterial PFTs, gut epithelial cells contract, thinning the epithelial layer while maintaining barrier function. This process involves massive actomyosin rearrangements and recovery of normal cytoskeletal morphology, via CyclinJ-dependent signaling [84]. Similarly, following PFT intoxication of HeLa cells in vitro, the actomyosin network recovers the normal organization with similar kinetics (~ 4 to 8 h), which correlates with the occasional release of large cytoplasmic containing blebs [80, 89]. Whether both phenomena are functionally interrelated is unknown, yet it is tempting to speculate that the mechanisms underlying actomyosin remodeling observed in vitro following PFT intoxication, not only favor PM repair, but also impact general epithelial organization and integrity during tissue responses to bacterial-mediated damage.

Microtubule (MT) dynamics also influences PM repair. Mechanically induced PM damage promotes calcium-dependent disassembly of MTs around the wound sites [159, 160] which facilitates PM repair [105, 135, 147, 161]. MT rearrangements assist continuous vesicle and lipid trafficking to the wound sites from the Golgi, protecting against recurrent mechanically induced PM wounds [160]. In line with this, translocation of Golgi-derived vesicles to the PM is important to reduce *M. tuberculosis*-induced damage and diminish infection [128]. Contrarily, repair of SLO-intoxicated cells depends on intact MTs as the addition of nocodazole, an established MTs disruptor, was proposed to inhibit PM repair [45]. However, PLY was shown to induce MT stabilization in a process that depends on Src kinase and may be linked to cell damage during infection [162].

### Cell survival pathways

Mitogen-activated protein kinases (MAPKs) are serine/threonine kinases that largely respond to stress stimuli and coordinate a variety of processes such as cell survival, metabolism, and proliferation [163]. Conventional MAPKs include the extracellular signal-regulated kinases 1 and 2 (ERK1/2), p38 isoforms (alpha, beta, gamma, and delta) and c-Jun amino (N)-terminal kinases 1, 2, and 3 (JNK1/2/3). Activation of MAPK pathways, in particular p38, is promoted by potassium efflux [40] and constitutes a widely conserved defense mechanism against PFT-mediated damage [4, 11, 164]. However, p38 activation is dispensable for the recovery of SLO-intoxicated keratinocytes [165]. p38 can be activated downstream the engagement of TLR4 receptor by different CDCs such as Anthrolysin (ALO) from *Bacillus anthracis*, which induces iNOS expression and promotes pro-inflammatory cytokines release [166, 167]. In parallel, LPS-mediated TLR4 stimulation activates p38 via the production of reactive oxygen species (ROS) and downstream the apoptosis signal-regulating kinase 1 (ASK1) activity [166, 167]. Whether such pathway is activated by PFTs is not known. However, during intoxication by alpha-toxin, ASK1 knockdown and ROS scavenging do not prevent p38 activation [165], suggesting that other factors may be involved.

During PFT intoxication, p38 activation is dependent on potassium efflux yet, how potassium depletion triggers MAPK signaling and which p38-regulated mechanisms promote cell recovery from PFT-induced damage remains unclear. Perturbations of potassium levels in different contexts such as low potassium diet [168] and skin wound healing [169] also trigger p38 activation. In addition, the decrease of cytosolic potassium levels activates the inflammasome NLRP3 [44] by a mechanism that involves the serine/threonine kinase Nek7, which regulates NLRP3 oligomerization and activation downstream of potassium efflux [170]. Interestingly, NLRP3 is activated during intoxication with the *Clostridium tetani* PFT, tetanolysin (TLO), and by *Streptococcus pyogenes* infection in an SLO-dependent manner, leading to caspase-1 activation and IL-1 beta release [171, 172]. However, the molecular details of a potential p38 and NLRP3 interconnection remain elusive.

To date, the activation of p38 was reported in response to all PFT tested—including *Clostridium perfringens* beta-toxin, aerolysin, alpha-toxin, VCC, HlyA, Cry toxins, and CDCs, such as PLY, SLO, LLO, ALO, Vaginolysin (VLY), and Inerolysin (INY) [40, 41, 50, 164, 165, 173–176]—in a variety of hosts including different mammalian cell lines, *C. elegans*, and various insect species [41–43, 177–180].

Activation of p38 and/or ERK1/2 induces the shedding of PM vesicles and receptors in microglia, platelets, and tumor cells [181–183]. Alpha-toxin- and ALO-induced p38 and ERK1/2 activation also promotes the shedding of cell adhesion and intracellular-contact molecules [124, 184]. Accordingly, MAPK-mediated PM shedding was thus proposed to contribute to tissue barrier disruption during PFT intoxication or PFT-mediated infection. Despite MAPK-mediated shedding of PFT pores was not yet identified, it is tempting to speculate that p38 activation could directly support PM repair by promoting the shedding of PFT pores. Indeed, p38 activity promotes recovery of potassium homeostasis and increases cell survival in response to PFT intoxication [41]. In addition, activation and transcriptional up-regulation of both p38 and JNK protect *C. elegans* from intoxication with Cry5B and large CDCs such as SLO [43, 179]. Both p38 and JNK regulate multiple downstream signals, which were shown to be protective upon PFT intoxication in vivo. Specifically, p38 up-regulates genes involved in the activation of the Unfolded Protein Response (UPR) and expression of putative cation efflux channels [42]. On the other hand, JNK was described as the master regulator of PFT-induced transcriptional responses, capable of up-regulating p38 specific pathways and innate immune signaling via the AP-1 transcription factor [179].

Reversely, MAPK activation during PFT intoxication may also have detrimental effects, as blocking p38 activity reduces PLY-mediated cytotoxicity in human SH-SY5Y neuroblastoma cells [185].

### ER stress and organelle damage

Perturbations on ER homeostasis lead to the accumulation of unfolded proteins, which are recognized by ER stress sensors that signal for the re-establishment of normal ER conditions. This response involves the: (1) reduction of general protein translation; (2) up-regulation of chaperone expression to enhance protein folding; (3) activation of ER degradation pathways [186]. This process is called Unfolded Protein Response (UPR) and comprises three main branches defined by the specific stress sensor engaged: inositol requiring enzyme 1 (IRE1), protein kinase RNA (PKR)-like ER kinase (PERK), and activating transcription factor 6 (ATF6) [186]. Under irreversible ER stress, the UPR can ultimately induce apoptosis [187–191].

PFTs activate IRE1 downstream p38-MAPK signaling in vivo, and both IRE1 and ATF6 protect *C. elegans* against intoxication by Cry5B [42], yet, the mechanisms through which UPR activation protects against PFT intoxication remain unclear. During infection, UPR activation by PFTs, limits intracellular replication of *L. monocytogenes* [187], or diminishes the growth of extracellular Group A *Streptococcus* through the production of specific metabolites [192]. In the other hand, UPR activation may also lead to cell death [193], particularly upon prolonged dysregulation of calcium signaling [37, 194]. During *S. pneumoniae* infection in vivo, circulating PLY targets cardiomyocytes, leading to uncontrolled activation of PKCα-troponin and UPR pathways, loss of the contractile properties, and acute cardiac injury [37].

Upon PFTs’ challenge, the perturbations of calcium homeostasis can be due to calcium influx from the extracellular environment, subsequent release of calcium by intracellular calcium stores, and, possibly, from direct damage of ER compartments, all contributing to ER stress [38, 195, 196]. In this context, different PFTs such as LLO, Aerolysin, and ShlA were shown to induce ER expansion, fission, and vacuolation [84, 89, 197]. PFT-mediated ER vacuolation may result from ER damage or cell death [84, 89]. Nevertheless, ER vacuolation in LLO-damaged cells ranges from mild fission to disruption of the entire ER network with cells recovering normal ER morphology following toxin washout [89]. ER fission likely limits ion and protein diffusion, and thus, this process could help cells isolating intracellular calcium pools, and preventing overwhelming and deleterious elevations. On the other hand, limiting protein diffusion may compromise host-cell signaling and affect the responses to intoxication and ultimately to infection.

The morphology of post-ER compartments and the Golgi apparatus is not affected by aerolysin even in cells with dramatic ER fission and vacuolation [197]. However, VCC-induced vacuoles recruit proteins from the trans-Golgi network (TGN46), and co-localize with late-endocytic (Rab7 and LAMP1) [198] and autophagic (LC3) compartments [199, 200]. Accordingly, both vacuole turnover and recovery of ER morphology following PFT intoxication may rely in autophagic pathways. In addition, cells respond to PFT-induced potassium efflux by inducing the formation of lipid droplets [41] and promoting caspase-1-dependent activation of sterol-responsive element-binding proteins (SREBPs) [201], central regulators of lipid metabolism, and membrane biogenesis [202]. Both processes appear to promote cell survival against PFT intoxication. Intriguingly, however, the host pore-forming protein gasdermin D, which is activated downstream caspase-1, up-regulates liver lipogenesis and contributes to the excess inflammation in mouse models of fatty liver disease [203]. Accordingly, it is possible that, while caspase-1-promoted fatty acid metabolism may increase cell-autonomous survival during PFTs’ intoxication, such mechanism may also trigger unexpected inflammatory processes in vivo.

Different PFTs can also directly target mitochondria [204–208], either affecting mitochondrial permeability, morphology, or functioning [204, 205, 207–211]. The *Helicobacter pylori* PFT, VacA, promotes Drp1-mediated mitochondrial fission and causes release of cytochrome c, an event also promoted by HlyA and the staphylococcal toxin Panton–Valentine leukocidin (PVL) [204–206, 208]. Mitochondrial damage caused by PVL and PLY also induces the release of Smac [206] and the apoptosis-inducing factor (AIF) [207] and mtDNA [209], respectively. Ultimately, this may lead to the activation of pro-apoptotic caspase-3 and -9, and trigger cell death [204, 206, 208]. Accordingly, inhibition of Drp1 was shown to prevent mitochondrial permeabilization and cell death upon VacA intoxication [212]. During infection, LLO-mediated transient mitochondrial fragmentation does not lead to cell death but promotes *L. monocytogenes* replication [213]. Contrarily, the PLY-mediated release of mtDNA within secreted microvesicles contributes to pro-inflammatory responses against *S. pneumoniae* [209].

PFT-induced lysosomal damage was also observed in epithelial cells during *L. monocytogenes* infection [214]. LLO and other CDCs induce lysosomal permeabilization and release of the lysosomal aspartyl-protease cathepsin D, which remains transiently active in the cytosol, but it is not involved in PFTs’ degradation [214]. LLO-dependent degradation of the crucial SUMOylation conjugation enzyme, Ubc9, promotes *L. monocytogenes* infection and is partially blocked by an aspartyl-protease inhibitor [215]. Impaired SUMOylation and alteration of the cellular proteome are promoted by LLO and PFO by decreasing the abundance of a variety of ubiquitilome-related proteins [216]. This process involves only post-transcriptional mechanisms and may also be beneficial for *L. monocytogenes* infection.

Altogether, PFT-induced organelle damage is linked to alterations of ion imbalance, cellular metabolism, and cell death. Nevertheless, in different model systems, cells have been shown to recover from such stress by coordinating different processes that may involve: rescuing pathways such as the UPR, putative calcium sequestration mechanisms, increased membrane synthesis and lipid metabolism, and purging or recycling of damage compartments by autophagy (Fig. 4).

### Autophagy

Autophagy constitutes a central defense mechanism against both extracellular and invading bacterial pathogens [12, 121, 217–219]. It is activated upon PFT-induced PM damage to sustain metabolic homeostasis as PM perforation causes nutrient and energy depletion. In particular, SLO, alpha-toxin and VCC, promote autophagy activation downstream AMPK or elF2alpha-kinase 4, known as energy- and nutrient-depletion sensors, respectively [220, 221]. Autophagy was also proposed to promote PM repair in *C. elegans* fed with *Escherichia coli* expressing Cry5B. The co-localization of Cry5B with autophagic markers suggests that autophagy participates in the degradation of PFTs [121]. In agreement, worms intoxicated with Cry5B, Cry21A, and SLO display decreased survival upon autophagy inhibition [121]. VacA stability is also promoted by the inhibition of autophagy [222]. VCC activates autophagy and induces the formation of large intracellular vacuoles, which are targeted by the autophagic machinery [199, 223]. In addition, inhibition of autophagy in VCC-intoxicated cells impairs cell survival [199]. Whether PFT-induced vacuoles are effectively removed by autophagy has never been confirmed. Nevertheless, autophagy plays a crucial role in ER remodeling [224], mitochondria degradation [225], and detection of damaged endosomes and lysosomes [226, 227]. Since PFTs promote ER vacuolation and can damage mitochondria and lysosomes, one can hypothesize that autophagy also protects intoxicated cells by removing damaged organelles (Fig. 4).

During infection, autophagy protects the host-cell cytosol against the activity of PFTs such as alpha-toxin and HlyA, which allow bacterial escape from intracellular vacuoles [12, 228], and limits VacA-induced cellular damage [222]. In contrast, the activation of autophagy during *L. monocytogenes* infection depends on LLO [229], but enables the formation of replication vacuoles (SLAPs—spacious *Listeria*-containing phagosomes), which may provide a niche for persistent infections [230, 231]. Thus, autophagy acts as an antimicrobial defense mechanism, but can also be exploited by PFT-expressing bacterial pathogens to promote their replication [232].

Although this review focuses on the mechanisms that protect host cells against PFTs, it is worth to mention that lytic concentrations or permanent exposure to PFTs cause irreversible PM permeability, culminating into uncontrolled necrotic cell death [11]. At sub-lytic doses, PFTs can induce different programmed cell death pathways, in particular pyroptosis and necroptosis, which significantly affect the outcome of infection in vivo [4, 11, 35, 64, 233, 234]. PFT-producing pathogens activate different cell death pathways [64]; however, the mechanisms underlying the activation of a specific cell death pathway during PFT intoxication or infection vary considerably according to the cell type or model organism and PFT tested. Whether the different cell death responses are either beneficial for the host—protecting it—or for the pathogen promoting infection needs further investigation.

## Open questions and future directions

PFTs were for long considered potent unsophisticated virulence factors whose solely function was to form PM holes in host cells. However, in the recent years, several studies revealed PFTs as multifaceted factors inducing a plethora of cellular responses. At sub-lytic concentrations, PFTs allow bacteria to manipulate host-cellular functions, not necessarily to kill the host cells but to promote overall pathogenesis [4, 235]. Studies performed in cultured cell lines in vitro generated an extensive amount of data but, paradoxically, failed to establish mechanisms through which sub-lytic concentrations of PFTs may promote systemic infections. Efforts should now be directed to further understand the complexity of PFTs activity and the host response during disease.

The cellular responses to sub-lytic concentrations of PFTs were assessed individually for specific PFTs, using several cultured cell lines and a range of different concentrations of purified toxins. Given that PFTs are widespread among bacterial pathogens and share essential functions and effects, in the future, PFTs should be studied together with an emphasis on common features. In particular, a combination between ultra-structural, super resolution, and live imaging microscopy analysis of wound sites is now important to characterize the architecture of PFT-mediated injuries and localized repair mechanisms. This approach could reveal broad antimicrobial strategies to target PFTs and fight several bacterial infections.

As described in this review, a multitude of mechanisms were involved in PFTs’ defense at the cellular level. Whether these mechanisms are part of a coordinated response regulated in time and space needs to be addressed. In addition, the interdependence of the different defense events and the molecular basis for their activation require further investigation. Importantly, findings in the context of PFTs response will be of interest in the other pathological contexts in which plasma membrane damage is occurring.

PFTs’ defense at the organism level also deserves further exploration. Indeed, the exact mechanisms of action of cellular defense pathways, where they act and how they are protective in a complex organism, remain mainly unidentified. Host specificity needs to be considered and future efforts should focus on the development and use of valuable infection models, such as *D. melanogaster*, *D. rerio,* and *C. elegans*, which are extremely versatile and for which a large number of molecular tools are available. The understanding of PFT-induced host barrier dysfunction, inflammation, and immune response disruption is of critical importance in the perspective of systemic infection, and needs to be analyzed in vivo in the context of organized tissues composed by a variety of cell types. In addition, emerging three-dimensional (3D) cell culture models may also represent valuable tools to study host responses to PFT activity in the context of tissue properties or organ complexity.

Altogether, these studies should help to find novel treatments targeting PFTs from a broad range of slightly different toxins or enhancing host defense mechanisms.

## Abbreviations

A: Annexin
ALG-2: Apoptosis-linked gene 2
ALIX: ALG-2-interacting protein X
ALO: Anthrolysin
ASM: Acid sphingomyelinase
ATF6: Activating transcription factor 6
CDC: Cholesterol-dependent cytolysin
ER: Endoplasmic reticulum
ESCRT: Endosomal sorting complex required for transport
INY: Inerolysin
IRE1: Inositol requiring enzyme 1
LLO: Listeriolysin O
MAPK: Mitogen-activated protein kinase
MLKL: Mixed lineage kinase-like
MT: Microtubule
mtDNA: Mitochondrial DNA
MVB: Multivesicular body
NMIIA: Non-muscle myosin IIA
NMII: Non-muscle myosin II
PERK: Protein kinase RNA (PKR)-like ER kinase
PFO: Perfringolysin O
PFT: Pore-forming toxin
PLY: Pneumolysin
PM: Plasma membrane
PVL: Panton–Valentine leukocidin
SLAPs: Spacious *Listeria*-containing phagosomes
SLO: Streptolysin O
Slp4-a: Synaptotagmin-like protein 4a
SNARES: *N*-ethylmaleimide sensitive factor attachment protein receptors
SREBPs: Sterol-responsive element-binding proteins
T3SS: Type-III secretion system
TLO: Tetanolysin
UPR: Unfolded protein response
VCC: *Vibrio cholerae* cytolysin
VLY: Vaginolysin
